# Risk practices and awareness of leptospirosis amongst residents of Zaria, Nigeria

**DOI:** 10.1038/s41598-024-66361-x

**Published:** 2024-07-02

**Authors:** Collins Chimezie Udechukwu, Caleb Ayuba Kudi, Paul Ayuba Abdu, Paul Habila Mamman, Nicholas Nathaniel Pilau, Kelvin Olutimilehin Jolayemi, Magdalene Ogbonneya Okoronkwo

**Affiliations:** 1 Department of Veterinary Medicine, Ahmadu Bello University, Zaria, Kaduna State Nigeria; 2 Department of Veterinary Microbiology, Ahmadu Bello University, Zaria, Kaduna State Nigeria; 3 Department of Veterinary Medicine, Usmanu Danfodiyo University, Sokoto, Sokoto State Nigeria; 4 Department of Veterinary Pharmacology and Toxicology, Ahmadu Bello University, Zaria, Kaduna State Nigeria

**Keywords:** Leptospirosis, Questionnaire, Risk factor, Zaria, Diseases, Health occupations, Risk factors

## Abstract

This study evaluated the level of risk practices and awareness of leptospirosis among residents of Zaria, Nigeria. A pre-tested questionnaires were administered via face-to-face interview to 100 residents. The data was analyzed using chi-square and multivariate analysis to identify risk factors for leptospirosis. The demography showed that the majority of the respondents were male, aged 21–40 years, and majorly crop farmers. The risk factors identified showed that males were 4.14 times more likely to be affected by leptospirosis (OR 4.14, 95% CI [1.93–5.37], *p* = 0.02) and the source of animal’s water was 5.56 times more likely to be contaminated by *Leptospira* spp. (OR 4.14, 95% CI [2.88–8.03], *p* = 0.01) and these relationships were significant. The majority of respondents were not aware of the disease (OR 1.87, 95% CI [1.22–4.57], *p* = 0.01) with 78% of the respondents not sure of which of the animal species leptospirosis affected (OR 1.67, 95% CI [1.07–2.62], *p* = 0.02). This study has demonstrated the existence of risk behaviors, and paucity of knowledge about leptospirosis in the study area. It is therefore recommended to organize an enlightenment program and the need for protective clothing for individuals occupationally at risk of infection by *Leptospira* spp.

## Introduction

Leptospirosis is an important zoonotic disease and poses a serious threat to public health and animal health worldwide. They represent about 70% of the number of emerging infectious diseases in recent times^1,2^. Humans living in high-risk areas, occupationally risk individuals such as farmers, veterinarians, and abattoir workers constitute a major group at risk of infection with *Leptospira* spp, due to the close contact that exists between them and animals/tissue or excreta in soil^2^. An estimated 320,000 occupationally related deaths from infectious diseases are reported yearly worldwide^3^. Occupation, migratory behaviour, gender, and age are all significant risk factors for leptospirosis^4^. In the past, leptospirosis was first considered as an occupational disease, whereby coal miners were the first occupational risk groups to be documented in the literature^5,6^.

Leptospirosis has a global distribution and is one of the leading causes of reproductive failure in cattle^7^. Tropical countries with significant rainfall and humidity, as well as marshy soil and paddy-growing areas, are more prone to the disease^8^. Leptospirosis is an infectious zoonotic disease of veterinary and public health importance caused by pathogenic bacteria belonging to the genus *Leptospira*. The disease is transmitted via contact with tissue or urine of infected animal reservoirs such as cattle, sheep, pigs, dogs, and rodents. Transmission occurs via direct contact of the mucous membrane or broken skin with the urine or tissue of infected animals or indirectly with a contaminated environment, especially water contaminated with the urine of infected animals^9,10^.

Therefore, an important factor affecting the risk of infection and the transmission of leptospirosis in Zaria Kaduna State, Nigeria is the attitude, knowledge, and practices of residents, especially occupationally risk individuals such as farmers, miners, hunters, veterinarians, and abattoir workers. Understanding the Knowledge and awareness of leptospirosis existence creates a platform for behavioural changes that will help in the prevention of leptospirosis^11^. This will also help in resource prioritization and dedication to the prevention and control of leptospirosis by the government^2^.

This study was therefore designed to assess the risk factors or practices by residents that can contribute to the transmission of leptospirosis as well as the level of awareness of leptospirosis in high-risk areas and individuals of Zaria, Kaduna State, Nigeria.

## Material and methods

### Study area

The study was conducted in Zaria and environs, Kaduna State, Nigeria. Dry season farming is the second most prevalent agricultural activity in Zaria with vegetables and cereals being the common produce and animal manure is used for farming and this could contribute to transmission of leptospirosis^12,13^. Zaria is characterized by a tropical climate of high temperature and high humidity almost all year round, with temperatures ranging between 30 and 36 ℃. Rainfall is fairly even for almost nine months in a year but typically heavier between July and August, with an annual rainfall of 1092.8 mm^12,13^.

### Identification of risk factors of leptospirosis in Zaria and its environs, Kaduna State, Nigeria

A pre-tested questionnaire containing both closed and open-ended questions was administered to 100 respondents in Zaria, Kaduna State, Nigeria willing to participate in the study using face interviews in eight (8) villages namely Bomo, Bassawa, Goruba, Panhauya, Ungwan Rimi, Hunkuyi, Shika and, Samaru within Zaria, Kaduna State, Nigeria. The questions were interpreted in the local dialect (Hausa language) of the respondents during the administration. The questionnaire was modified according to new variables that were encountered during pre-testing. The questionnaire variable covered information on the knowledge, attitude, practice of respondents to leptospirosis, exposure, and sources of transmission and occupationally at-risk individuals. The questionnaire was administered to farm owners, feed millers, grain sellers, feed store owners, farm managers, farm attendants, and residents through interviews during on-site visits. Personal observation of farm activities, premises, and houses was used to validate residents’ responses.

### Ethics approval and consent to participate

All procedures performed in the study were under the Declaration of Helsinki. The study protocols were approved by the Ahmadu Bello University Ethical Committee (ABUCAUC/2021/109). All adult participants provided written notification of consent before participating in the study.

### Data analysis

Discrete data obtained from the questionnaire were entered into Microsoft Excel® sheets and the frequencies were converted to percentages and presented in the form of tables. Chi-square and multivariate analysis were used to check for association between variables. Statistical Package for Social Science (SPSS) version 21 was used for data analysis, values of *p* < 0.05 were considered significantly different.

## Results

### Demographic features of respondents

The demography showed that the majority of the respondents were male, aged 21–40 years, and majorly crop farmers (Table 1).

**Table 1:** Demography of respondents.

Parameteres	Frequency	Odds ratio	95% Confidence interval	p-value
Age of respondents (years)				
0–20	10	1.67	0.92–3.02	0.28
21–40	59	1.80	1.34–2.42	0.00*
41–60	25	1.70	0.73–3.96	0.08
61 and above	6	Ref	–	–
Occupation				
Crop farmer	60	2.51	1.41–4.45	0.00*
Livestock farmer	21	Ref	-	–
Grain store owner	4	0.66	0.28–1.54	0.80
Sex				
Male	67	4.14	1.93–5.37	0.02*
Female	33			

*Value of *p* ≤ 0.05 is considered significant.

### Risk factors

The results showed that the major risk factors observed in the study area were sex, occupation, outdoor activities around water bodies, flooding, and consumption of rodents. The risk factors identified showed that males were 4.14 times more likely to be affected by leptospirosis (OR 4.14, 95% CI [1.93–5.37], *p* = 0.02) and the source of animal water was 5.56 times more likely to be contaminated by *Leptospira* spp. (OR 4.14, 95% CI [2.88–8.03], *p* = 0.01) and these relationships were significant. Crop farmers were 3.21 times more likely to be infected by *Leptospira* spp. in comparison to livestock farmers (OR 3.21, 95% CI [1.36–5.42], *p* = 0.00) (Table 2).

**Table 2:** Risk factors associated with leptospirosis.

Question	Frequency	Odds ratio	95% Confidence interval	*p*-value
How is leptospirosis transmitted?				
Contact with rodents	16	1.23	0.56–2.69	0.59
Fomites	0	0.00	–	–
Airborne	0	0.00	–	–
Waterborne	2	0.32	0.06–1.64	0.17
Coitus	0	0.00	–	–
Feed	2	0.32	0.06–1.64	0.17
Don’t know	5	2.18	0.65–7.30	0.21
What is the source of animal drinking water?				
Tap	8	5.56	2.88–8.03	0.01*
Well	43			
Sex				
Male	67	4.14	1.93–5.37	0.02*
Female	33			
Occupation				
Crop farmer	60	3.21	1.36–5.42	0.00*
Livestock farmer	21			
Over the past 6 months, have you experienced flooding around your houses, farms, stores, and workplaces?				
Yes	41	0.48	0.34–1.05	0.28
No	59			
How do you dispose of animal dung?				
Empty into river	1	–	–	–
Burn	2	–	–	–
Dump in community waste site	30	Ref	–	–
Dump around house	34	1.17	0.56–2.46	0.60
Dump in the bush	16	0.55	0.25–1.21	0.14

*Value of *p* ≤ 0.05 is considered significant.

### Knowledge, attitude, and practice

The majority of respondents were not aware of the disease (OR 1.87, 95% CI [1.22–4.57], *p* = 0.01) with 78% of the respondents not sure of which of the animal species leptospirosis affect (OR 1.67, 95% CI [1.07–2.62], *p* = 0.02) and 80% of respondents don’t know how leptospirosis is being transmitted (OR 2.08, 95% CI [1.34–3.24], *p* = 0.002). Baiting rodents were common practice of the respondents in the study area with a 3.64 times likelihood of baiting rodents compared to doing nothing (OR 3.64, 95% CI [1.78–6.30], *p* = 0.01) (Table 3).

**Table 3:** Knowledge, attitude and practice of respondents.

Question/responses	Frequency	Odds ratio	95% Confidence interval	*p*-value
Have you heard of leptospirosis?				
Yes	25	1.87	1.22–4.57	0.01*
No	71	Ref		
Not applicable	4			
What species of animal does it affect?				
Cattle	10	0.76	0.47–1.24	0.28
Sheep	3	0.41	0.10–1.73	0.23
Goat	3	0.41	0.10–1.73	0.23
Dog	2	–	–	–
Human	4	0.53	0.14–2.01	0.35
Don’t know	78	1.67	1.07–2.62	0.02*
Do you see rodents around your houses, farms, stores, and workplaces?				
Yes	93	13.35	4.29–41.21	0.01*
No	7	Ref		
If yes, what did you do?				
Bait them	77	3.64	1.78–6.30	0.01*
Nothing	23	Ref		
Do you consider leptospirosis as a differential diagnosis in disease management?				
Yes	36	0.68	0.30–1.54	0.35
No	11			
Not applicable	53	Ref		
How is leptospirosis transmitted?				
Contact with rodents	16	0.77	0.45–1.33	0.36
Fomites	0	–	–	–
Airborne	0	–	–	–
Waterborne	2	–	–	–
Coitus	0	–	–	–
Feed	2	–	–	–
Don’t know	80	2.08	1.34–3.24	0.002*
Over the past 6 months, have you done any outdoor activities around water?				
Yes	53	2.01	0.89–4.34	0.10
No	47	Ref		
Over the past 6 months, have you experienced flooding around your houses, farms, stores, and workplaces?				
Yes	41	2.13	0.94–5.61	0.19
No	59	Ref		

*Value of *p* ≤ 0.05 is considered significant.

## Discussion

The risk factors for leptospirosis in humans identified in the study area were middle-aged males between the ages of 21 to 40 years. Many of them were crop farmers (60%) and were not aware of leptospirosis. Also, 36% were animal health professionals who did not consider leptospirosis as a differential diagnosis of the disease. The finding agrees with the report of Ngbede^2^ who also highlighted that the majority of the respondents in the study area were not aware of leptospirosis and this puts them at risk of infection. Precautionary steps to prevent infection by the organism are often neglected by Zaria locals, as the majority of those surveyed were unaware of the disease. Lack of formal education by respondents in the study area as well as the lack of awareness of the disease can be linked to the absence of a local name for the disease in the study area^2^. Also, the reason for this finding on middle-aged males may not be unconnected with the fact that the study area had lots of persons within the age group of 21 to 40 years, also in their productive years of life, and were mostly crop farmers who were always in direct exposure to outdoor settings of farms and water bodies possibly contaminated with rodent urine. Furthermore, despite Udechukwu^14^ establishing a high prevalence in the study area, there may have been insufficient active surveillance of *Leptospira* spp. by local public health authorities and researchers within the study location, which could have contributed to the lack of awareness about leptospirosis in this study. We postulated that the nonspecific clinical signs and the possibility of leptospirosis mimicking other febrile illnesses may be responsible for the low level of consideration of leptospirosis as a differential diagnosis by clinicians. The findings on the occupation and educational status of the respondents agree with the report of Buchanan^5^ who stated that occupation, education, gender, and age are significant risk factors for leptospirosis. This study showed that males are at higher risk of infection than females as the males in the study location were more prone to occupational exposure and outdoor exposure than females^15^. This may be connected with the sociocultural and religious practices in some parts of Northern Nigeria where uneducated young women are not allowed to either interact or work outside their homes with their husbands^16^. The results are in agreement with the report of the Leptospira Burden Epidemiology Reference Group^15^ which states that men are likely to become more infected with *Leptospira* spp, than women as they are more prone to exposure in outdoor settings.

Activities near water bodies, flooding, and rodent proliferation were also highlighted in this study as risk factors in the study area. This may have been because of the unhygienic practices that have been observed in the area, which may have increased the likelihood that water bodies within the study area will become contaminated during rainfall and flooding^17^, leading to an increase in the prevalence of *Leptospira* spp. Water from the well is the major source of drinking water in the study area and can easily be contaminated with flooded water contaminated with rodent urine, with consequent increase in the risk of *Leptospira* spp. burden in the study area as also reported by WHO^15^.

## Conclusion

This study has shown that the residents of Zaria, Kaduna State Nigeria are at risk of infection with *Leptospira* spp. due to the low level of awareness of the disease. It is therefore recommended to organize an enlightenment program on the implication of contact with animals/or their tissues without adequate protection and the need for the use of such protective clothing for individuals occupationally at risk of infection by *Leptospira* spp.

## Data Availability

The dataset used and/or analyzed during the current study is available from the corresponding author upon reasonable request.
